# Exploring the role of epileptic focus lateralization on facial emotion recognition in the spectrum of mesial temporal lobe epilepsy

**DOI:** 10.3389/fnsys.2024.1491791

**Published:** 2025-01-06

**Authors:** Fabio Iannaccone, Chiara Pizzanelli, Francesca Lorenzini, Francesco Turco, Chiara Milano, Claudia Scarpitta, Luca Tommasini, Gloria Tognoni, Riccardo Morganti, Enrica Bonanni, Gabriele Siciliano

**Affiliations:** ^1^Neurology Unit, Department of Clinical and Experimental Medicine, University of Pisa, Pisa, Italy; ^2^Department of Clinical and Experimental Medicine, Section of Statistics, University of Pisa, Pisa, Italy

**Keywords:** epilepsy, mesial temporal lobe epilepsy, facial emotion recognition, asymmetry, social cognition, age at epilepsy onset, mesial temporal sclerosis, febrile seizures

## Abstract

**Introduction:**

Evidence increasingly shows that facial emotion recognition (FER) is impaired in refractory mesial temporal lobe epilepsy (rMTLE), especially in patients with a right focus. This study explores FER in both mild (mMTLE) and refractory forms, examining the influence of epileptic focus lateralization on FER.

**Methods:**

50 MTLE patients, categorized by epilepsy severity and focus lateralization, were compared with healthy controls. FER was assessed using the Ekman Faces Test (EFT), which evaluates recognition of six basic emotions, alongside a battery of cognitive and mood tests.

**Results:**

mMTLE patients showed selective deficits in recognizing fear and anger, while rMTLE patients displayed broader deficits, affecting all emotions except surprise. Patients with a right focus underperformed across all negative emotions, whereas those with a left focus showed deficits mainly in fear and anger. Analysis indicated that early epilepsy onset was associated with poorer FER in right-focused patients; febrile seizures and mesial temporal sclerosis significantly impacted FER in left-focused patients.

**Conclusion:**

MTLE affects circuits of FER even in mild subjects, although to a lesser extent than in refractory ones. Earlier onset of MTLE could disrupt the development of FER, possibly interfering during a critical phase of maturation of its circuits, when the focus is right. Conversely, left MTLE may cause less damage to FER circuits, requiring additional factors such as a history of febrile seizures and/or mesial temporal sclerosis for significant impact. Clinically, refractory and right-sided MTLE might be viewed as risk factors of FER deficits.

## 1 Introduction

Epidemiological studies have shown that people with epilepsy often face challenges in forming effective interpersonal connections, leading to diminished job opportunities and unstable emotional and family relationships (Steiger and Jokeit, 2017; Beghi et al., 2019; Jasionis et al., 2021). Beyond social stigma, evidence suggests epilepsy itself may contribute to social dysfunction (Broicher et al., 2012). Social cognition—the ability to construct and flexibly use representations of the relationships between oneself and others to guide social behavior (Adolphs, 2001)—is crucial in these dynamics. Among the objective measures of social cognition, facial emotion recognition (FER)—the ability to identify basic emotions from facial expressions—has been extensively studied in various neurological disturbances (Marco-Garcia et al., 2019), including epilepsy (Edwards et al., 2017; Bora and Meletti, 2016).

In temporal lobe epilepsy (TLE), the most common type of focal epilepsy in adults, FER is consistently compromised, especially in recognizing negative emotions such as anger, fear, sadness, and disgust, while deficits in positive emotions like happiness and surprise are less marked (Broicher et al., 2012; Edwards et al., 2017; Meletti et al., 2003; Hennion et al., 2015). However, clinical variables influencing FER performance remain unclear. Factors such as duration of epilepsy, seizure frequency, and number of antiseizure medications (ASMs) do not consistently correlate with FER deficits (Edwards et al., 2017; Bora and Meletti, 2016). In contrast, earlier age at epilepsy onset reliably predict poorer FER outcomes (Meletti et al., 2003, 2009), possibly due to interference with the maturation of FER capabilities, which undergo significant changes from childhood to adulthood (Rodger et al., 2015).

Lateralization of the epileptic focus in TLE also appears to play a role; right focus may more significantly impair FER, affecting both severity and range of affected emotions (Bora and Meletti, 2016; Meletti et al., 2003, 2009; Benuzzi et al., 2004), underscoring the right hemisphere’s predominant role in emotion recognition (DeKosky et al., 1980; Gainotti, 1983; Natale et al., 1983).

While there is substantial evidence of FER impairment in patients with TLE, consensus on its clinical determinants is lacking, likely due to small, heterogeneous samples where different TLE types and severities are grouped together (Edwards et al., 2017; Bora and Meletti, 2016; Tanaka et al., 2013). To our knowledge, no studies have specifically focused on mild forms of mesial temporal lobe epilepsy (MTLE), nor combined MTLE severity with focus lateralization.

Given this background, we aimed to: (i) determine whether MTLE impairs FER despite excellent seizure control; (ii) compare FER profiles in mild MTLE (mMTLE) versus refractory MTLE (rMTLE); (iii) assess FER differences based on focus lateralization; and (iv) identify clinical variables predicting worse FER performance in MTLE.

## 2 Methods

### 2.1 Participants

Fifty MTLE patients (25 with mMTLE and 25 with rMTLE) were consecutively recruited at the Center for Diagnosis and Treatment of Epilepsy—Neurology Unit of the Azienda Ospedaliero-Universitaria Pisana. Diagnosis of MTLE was based on International League Against Epilepsy (ILAE) classification of seizures and epilepsy (Fisher et al., 2014; Scheffer et al., 2017), and on clinical ictal features, i.e., seizures with manual and oroalimentary automatisms or prominent experiential phenomena, gustatory or olfactory hallucination, eventually followed by arrest and unresponsiveness (Fisher et al., 2017). All patients underwent 3-Tesla brain magnetic resonance imaging (MRI) to evaluate the presence of abnormalities involving mesial temporal lobes, mainly mesial temporal sclerosis (MTS). Multiple scalp video-EEG recordings were employed to detect ictal and/or interictal EEG abnormalities. Patients with clinical or EEG features consistent with a possible extratemporal involvement were excluded from this study.

Patients were categorized as rMTLE, if they continued to experience seizures despite at least two adequate trials of ASMs, according to the ILAE definition (Kwan et al., 2010), and as mMTLE if they experienced at least 24 months of seizure freedom, with or without ASMs, according to the definition of Labate et al. (2011). Additionally, patients were categorized by the lateralization of their temporal focus—right, left, bilateral, or unknown—determined through lateralized EEG discharges, seizure features, and/or MRI findings. Twenty-five healthy subjects were recruited as controls (HC). All the subjects in mMTLE, rMTLE and HC groups were right-handed. We collected demographic and clinical data for all the participants: demographic features included sex, age and schooling; clinical features included age at epilepsy onset, epilepsy duration, seizures frequency, number of ASMs, history of febrile seizures (FSs) and type of temporal lobe lesion (if present). The study was approved by the Research Ethic Committee of Azienda Ospedaliero-Universitaria Pisana and it was carried out according to the Helsinki Declaration.

### 2.2 Facial emotion recognition and neuropsychological assessment

FER was assessed through the Ekman 60 Faces Test (EFT), which evaluates the ability to recognize six basic emotions (happiness, surprise, anger, disgust, fear, and sadness) from facial expressions. It comprises 60 images (10 faces per emotion) displayed for 5 s each. One point is assigned for each correct answer, for a maximum score of 60, adjusted based on sex, age, and schooling. Additionally, six separate sub-scores are calculated, one for each emotion. Before enrollment, all participants completed the Brief Intelligence Test (Sartori et al., 1995) and the Montreal Cognitive Assessment (Santangelo et al., 2015) to assess cognitive function. Patients also underwent a psychiatric interview and completed the Hospital Anxiety and Depression Scale (HADS) (Costantini et al., 1999) and the Toronto Alexithymia Scale-20 (TAS-20) (Bressi et al., 1996). Exclusion criteria were: (i) seizure onset outside mesial temporal structures; (ii) abnormal scores in cognitive assessments; (iii) psychiatric comorbidities.

### 2.3 Statistical analysis

Categorical data were described with absolute and relative (%) frequency, continuous data were summarized with mean and standard deviation. The normality of distributions was evaluated by Kolmogorov–Smirnov test. Successively, to compare categorical and continuous data with the group variable chi-square test and *t*-test for independent samples or one-way ANOVA, followed by multiple comparisons with Bonferroni method, were performed. To assess the linear relationships between the EFT scores and disease-related factors (age at epilepsy onset, epilepsy duration, annual seizure frequency and number of ASMs) Pearson’s correlation analysis was applied. To determine the independent contributions of each disease-related factor, multiple linear regression was used as multivariate model. Finally, a mediation analysis was performed to examine whether epilepsy duration mediates the relationship between age at epilepsy onset and EFT scores. The significance level was set at 0.05 and all analyses were carried out using SPSS v.29.

## 3 Results

### 3.1 Clinical and demographic features

Participants’ features are illustrated in Table 1. There were no statistically significant differences in sex (*p* = 0.947), age (*p* = 0.205) or schooling (*p* = 0.078) among mMTLE, rMTLE and HC. Regarding disease-related factors, the rMTLE group showed a statistically significant lower age at epilepsy onset (*p* = 0.015), a longer duration of epilepsy (*p* < 0.001), a higher number of ASMs (*p* < 0.001), and a higher annual seizure frequency (*p* < 0.001). There were no statistically significant differences in sex (*p* = 0.549), age (*p* = 0.612), schooling (*p* = 0.355), age at epilepsy onset (*p* = 0.940), epilepsy duration (*p* = 0.639), annual seizure frequency (*p* = 0.959), number of ASMs (*p* = 0.794), history of febrile seizures (*p* = 0.549), temporal lobe lesions (*p* = 0.249) or MTS (*p* = 0.724) between patients with right and left focus. All the included subjects exhibited normal scores in cognitive assessment, HADS and TAS-20.

**Table 1:** Clinical and socio-demographic characteristics of the study population stratified for group.

	HC (*n* = 25)	mMTLE (*n* = 25)	rMTLE (*n* = 25)	*p* values
Sex				
M (%)	11 (44%)	11 (44%)	10 (40%)	0.947
F (%)	14 (56%)	14 (56%)	15 (60%)	
Age, y, mean ± SD	50.64 ± 16.31	49.00 ± 15.14	56.24 ± 13.12	0.205
Schooling, y, mean ± SD	14.32 ± 2.80	13.80 ± 4.48	12.00 ± 3.74	0.078
Age at epilepsy onset, y, mean ± SD		32.24 ± 14.86	20.60 ± 17.68	**0.015**
Epilepsy duration, y, mean ± SD		16.72 ± 14.03	35.68 ± 21.71	**< 0.001**
Annual seizure frequency, mean ± SD		0	30.00 ± 62.00	**< 0.001**
Number of ASMs, mean ± SD		1.52 ± 0.71	3.24 ± 1.20	**< 0.001**
History of febrile seizures (%)		4 (16%)	9 (36%)	0.107
Cryptogenic Epilepsy (%)		14 (56%)	9 (36%)	0.156
Temporal lobe lesions		11 (44%)	14 (64%)	0.396
Mesial temporal lobe sclerosis (%)		8 (32%)	9 (36%)	–
Dermoid cyst (%)		0	1 (4%)	–
Astrocytoma (%)		0	1 (4%)	–
Cavernous angioma (%)		0	1 (4%)	–
Encephalitis/Meningitis (%)		1 (4%)	1 (4%)	–
DNET (%)		2 (8%)	1 (4%)	–
Epileptogenic focus lateralization				
Right		11 (44%)	10 (40%)	0.774
Left		6 (24%)	7 (28%)	0.747
Bilateral		0	7 (28%)	–
Unknown		8 (32%)	1 (4%)	–

M, Males; F, Females; SD, Standard Deviation; y, years; HC, Healthy Controls; mMTLE, mild Mesial Temporal Lobe Epilepsy; rMTLE, resistant Mesial Temporal Lobe Epilepsy; ASMs, Anti Seizures Medications; DNET, Dysembryoplastic Neuroepitelial Tumor. Bold values indicate statistical significance.

### 3.2 Ekman faces test: the impact of drug-resistance and epileptic focus lateralization

The mean scores for EFT are summarized in Table 2. EFT total score was significantly impaired in both rMTLE (*p* < 0.001) and mMTLE (*p* = 0.002) patients when compared to HC. In the rMTLE group, FER was significantly compromised in happiness (*p* = 0.006), fear (*p* < 0.001), disgust (*p* = 0.005), anger (*p* = 0.004) and sadness (*p* = 0.002), while no significant difference was found in surprise recognition when compared to HC. Patients with mMTLE showed impairment only in recognizing fear (*p* < 0.001) and anger (*p* = 0.013). When comparing the two patient subgroups, the rMTLE group exhibited statistically lower scores only in the recognition of disgust compared to the mMTLE group.

**Table 2:** Results for each group on EFT.

Ekman 60-Faces Test (EFT)	HC (*n* = 25)	mMTLE (*n* = 25)	rMTLE (*n* = 25)	ANOVA		Bonferroni multiple comparisons			
							mMTLE vs. HC	rMTLE vs. HC	rMTLE vs. mMTLE
Total score, mean ± SD	50.81 ± 4.04	44.62 ± 5.88	41.46 ± 7.88	F	15.018	MD	6.196	9.357	3,160
				*p*	**<0.001**	*p*	**0.002**	**<0.001**	0.219
Surprise, mean ± SD	9.28 ± 0.93	9.12 ± 1.01	8.28 ± 2.22	F	3.153	MD	0.160	1.000	0.840
				*p*	**0.049**	*p*	1.000	0.067	0.160
Happiness, mean ± SD	10	9.92 ± 0.27	9.72 ± 0.45	F	5.442	MD	0.080	0.280	0.200
				*p*	**0.006**	*p*	1.000	**0.006**	0.075
Fear, mean ± SD	7.60 ± 2.00	4.20 ± 2.97	3.08 ± 2.43	F	22.171	MD	3.400	4.520	1.120
				*p*	**<0.001**	*p*	**<0.001**	**<0.001**	0.353
Disgust, mean ± SD	8.40 ± 1.52	8.04 ± 1.67	6.72 ± 2.17	F	5.967	MD	0.360	1.680	1.320
				*p*	**0.004**	*p*	1.000	**0.005**	**0.036**
Anger, mean ± SD	7.76 ± 1.71	6.24 ± 1.83	6.04 ± 1.92	F	6.634	MD	1.520	1.720	0.200
				*p*	**0.002**	*p*	**0.013**	**0.004**	1.000
Sadness, mean ± SD	8.36 ± 1.25	7.72 ± 1.74	6.44 ± 2.58	F	6.349	MD	0.640	1.920	1.280
				*p*	**0.003**	*p*	0.742	**0.002**	0.067

EFT, Ekman 60-Faces Test; HC, Healthy Controls; mMTLE, mild Mesial Temporal Lobe Epilepsy; rMTLE, resistant Mesial Temporal Lobe Epilepsy; ANOVA, Analysis of Variance; SD, Standard Deviation; F, ANOVA F-value; *p*, *p*-value; MD, Mean Difference. Bold values indicate statistical significance.

**Figure 1:**
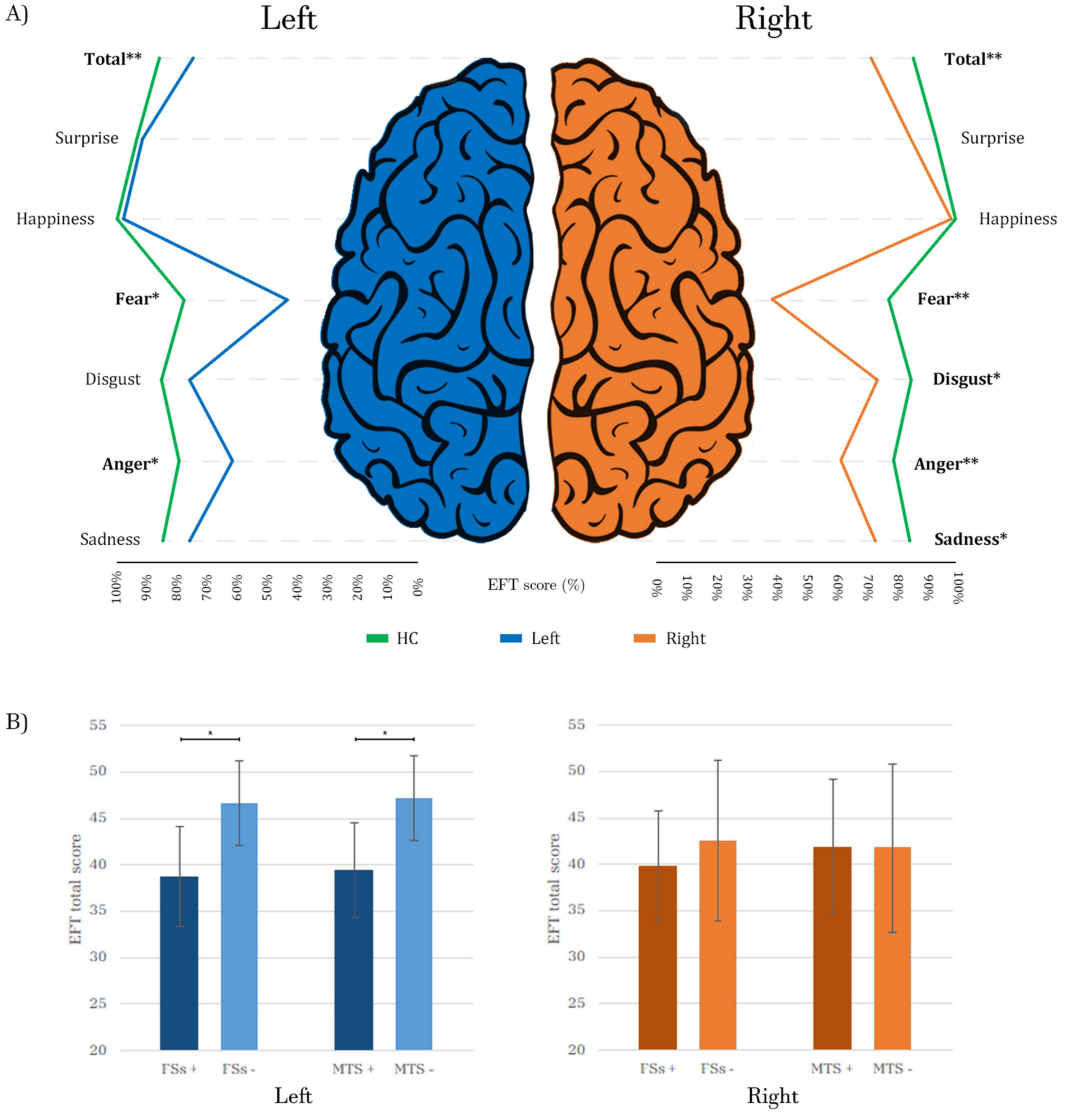
**(A)** Comparison of EFT scores between patients with right (*n* = 21) and left (*n* = 13) epileptic foci vs. HC (*n* = 25). Each score is represented as a percentage of the maximum (60/60 for the total score and 10/10 for each emotion). Different colors of the lines represent the three subgroups of subjects: the green lines depict the performance of HC, while the blue and orange lines represent the scores of left and right patients, respectively. The lines for the two patient groups are below those of the HC, following a similar pattern for both right and left foci, with notable dips at fear and anger, where the differences from HC are more pronounced, especially for the right patients. **(B)** Impact of FSs and MTS on the EFT total score in right and left patients: both variables are associated with statistically significant lower scores in patients with a left focus, while no significant differences are observed in patients with a right focus. HC, Healthy Controls; Total, EFT total score; Left, patients with left epileptic focus; Right, patients with right epileptic focus; FSs+, patients with Febrile Seizures; FSs−, patients without Febrile Seizures; MTS+, patients with Mesial Temporal Sclerosis; MTS−, patients without Mesial Temporal Sclerosis; *p ≤ 0.05; **p ≤ 0.001. Bold values indicate statistical significance.

Out of the 50 patients, 21 had a right-sided focus, 13 had a left-sided focus, and 7 had bilateral MTLE (9 unknown). The left MTLE group had the highest EFT total score (43.61 ± 6.12 (SD)) followed by the right MTLE group (41.79 ± 7.95), while the bilateral MTLE group had the lowest score (39.64 ± 7.02). However, an ANOVA revealed no significant differences in EFT total scores among the three MTLE groups (*p* = 0.504). In comparisons to HC, right-focus patients exhibited significantly more severe and widespread impairments, with lower EFT total scores (*p* < 0.001) and lower sub-scores for fear (*p* < 0.001), disgust (*p* = 0.041), anger (*p* = 0.001) and sadness (*p* = 0.045). Conversely, left-focus patients, in comparisons to HC, exhibited impairments in EFT total scores (*p* < 0.001) and in sub-scores for fear (*p* = 0.003) and anger (*p* = 0.002) (Figure 1A).

When considering both drug resistance and side of the focus, a trend emerged: the worst scores were obtained by resistant patients with bilateral focus (39.64 ± 7.02), followed by right rMTLE (41.62 ± 9.57), right mMTLE (41.94 ± 6.64), left rMTLE (42.36 ± 7.31) and left mMTLE (45.07 ± 4.57). The best scores were obtained by HC (50.82 ± 4.04). However, an ANOVA analysis (*p* < 0.001) with Bonferroni multiple comparisons revealed significant differences only between HC and bilateral (*p* = 0.002), right rMTLE (*p* = 0.004), right mMTLE (*p* = 0.004), and left rMTLE (*p* = 0.039). No statistically significant difference was found between HC and left mMTLE (*p* = 0.740) (Supplementary Figure S1).

### 3.3 Correlations with clinical variables

**Figure 2:**
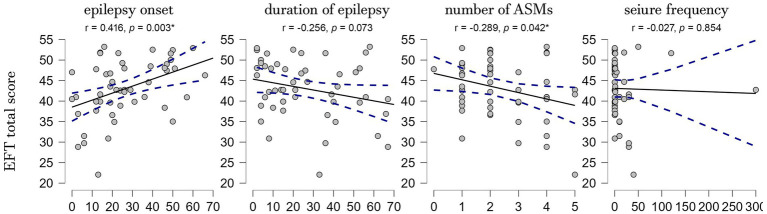
Scatterplots showing the correlations between the EFT total score and the clinical variables. EFT, Ekman Faces Test; r, Pearson correlation coefficient; *p*, *p*-value; *p ≤ 0.05.

Pearson’s correlation analysis revealed a significant association between EFT total score and the number of ASMs (r = −0.289, *p* = 0.042) and age at epilepsy onset (r = 0.416, *p* = 0.003), while no significant correlations were found with the duration of epilepsy (r = −0.256, *p* = 0.073) and seizure frequency (r = −0.027, *p* = 0.854). A multiple linear regression analysis showed a positive correlation only with the age at epilepsy onset (r = 0.492, *p* = 0.012) (Figure 2). To further investigate the relationship between age at epilepsy onset, duration of epilepsy, and FER performance, we conducted a mediation analysis. This analysis revealed that the direct effect of age at onset on FER performance was significant (estimate = 0.197, *p* = 0.009), while the indirect effect through duration was not significant (estimate = −0.026, *p* = 0.633). When the multiple linear regression analysis was conducted separately for patients with right and left epileptic foci, the association between age at epilepsy onset and FER performance persisted only in the right-focus group (*p* = 0.032).

A history of FSs during childhood was significantly associated with lower EFT total scores across the entire patient cohort (*p* = 0.008) and in patients with left focus (*p* = 0.016), but not in those with a right focus (*p* = 0.476). The presence of temporal lobe lesions did not correlate with lower scores either in the entire patient group (*p* = 0.172), or among those with right (*p* = 0.584) or left (*p* = 0.134) foci. However, MTS was linked to lower scores only in subjects with a left focus (*p* = 0.014), but not in the entire patient group (*p* = 0.127) nor in subjects with a right focus (*p* = 0.971) (Figure 1B).

## 4 Discussion

This study investigated FER across the MTLE spectrum, evaluating mMTLE separately from rMTLE and assessing differences based on focus lateralization. We used the Ekman 60 Faces Test to assess recognition of six basic emotions. Our main findings are: (i) mMTLE patients showed selective deficits in fear and anger recognition compared to HC, while rMTLE had broader deficits, affecting the recognition of all basic emotions except surprise; (ii) when categorizing subjects based on the side of the epileptic focus, patients with right MTLE underperformed in all negative emotions—fear, disgust, anger, and sadness—while those with a left focus demonstrated selective impairments in fear and anger; (iii) among the clinical variables, an earlier onset of epilepsy was associated with poor FER performance in patients with a right focus, but not in patients with a left focus; moreover, a history of FSs and the presence of MTS were linked to worse FER performance only in patients with a left focus.

### 4.1 Drug-sensitivity and lateralization of epileptic focus

Previous research on FER in TLE involved drug-resistant patients or mixed case series, often lacking precise clinical characterization (Broicher et al., 2012; Hennion et al., 2015; Tanaka et al., 2013). Generally, TLE patients have been found to be compromised in recognizing negative emotions (anger, fear, sadness, and disgust), and to a lesser extent, positive emotions (happiness and surprise) (Bora and Meletti, 2016; Qi et al., 2022). For instance, Reynders et al. (2005) found poor total FER and fear scores in 27 patients with drug-resistant TLE. Similarly, Walpole et al. (2008) reported low EFT scores in 16 drug-resistant TLE patients. Additionally, Tanaka et al. (2013) observed low total, fear and disgust scores in 63 MTLE patients who were poorly described in terms of clinical characteristics. Hennion et al. (2015) and Broicher et al. (2012) also found impairments in fear and disgust among mixed TLE patients, where the reported clinical features (seizure frequency and number of ASMs) could not clarify proportions of mild and refractory patients. The most extensive and precisely characterized cohort was described by Meletti et al. (2009), who found significant impairments across all negative emotions in FER (sadness, fear, disgust, and anger) in 140 drug-resistant MTLE patients.

Our mMTLE group resulted selectively compromised in fear and anger. They showed lesser impairment compared to Meletti’s resistant group and to our own rMTLE group. Notably, our study employed a more challenging version of the EFT compared to the one used by Meletti, featuring 6 emotions (including surprise) instead of 5, 10 faces per emotion rather than 5, and a picture viewing time of only 5 s rather than no time limit. This is reflected in our HC’s average total EFT score of 50.81, translating to 84.7% of the maximum score, in contrast to Meletti’s HC, where the correct recognition rate was 96.4%. Consequently, our test may be considered more sensitive in detecting deficits, which accounts for the generally lower performances observed across all subjects examined. As in previous studies, our study revealed a more pronounced impairment in the recognition of negative emotions, even affecting the mild patient population who, despite excellent seizure control and favorable course of the disease, scored between HC and rMTLE patients, without being statistically different from the refractory group.

Regarding the lateralization of the epileptic focus, comparisons of emotion-specific sub-scores between patients with right and left foci and HC revealed more extensive emotional involvement among right-sided patients (fear, disgust, anger, sadness) compared to left-sided patients (only fear and anger). These observations are consistent with literature suggesting more severe impairment in patients with right-MTLE compared to left-MTLE (Meletti et al., 2009; Golouboff et al., 2008; Kuchukhidze et al., 2021), supporting the notion that the right hemisphere plays a predominant role in the recognition and processing of emotions (DeKosky et al., 1980; Gainotti, 1983; Natale et al., 1983).

It is fascinating to note that the two subgroups showing better FER performance—mild and left MTLE—failed to recognize only fear and anger among the 6 basic emotions. These expressions, often the most commonly impaired in TLE as reported in the literature (Edwards et al., 2017; Bora and Meletti, 2016), are sometimes classified as ‘threatening’ because they help an individual identify and avoid potential threats, a trait that seems to play a central role in the evolutionary history of mammals (Juncai et al., 2017). This observation is reinforced by a study by Kobiella et al. (2008), which found that 7-month-old babies could discern the social meanings expressed by faces showing anger and fear, rather than simply recognizing them as negative stimuli. Expressions of fear and anger, more so than others, might activate specific neural circuits that are also implicated in MTLE. This could explain why these emotions are selectively compromised, while others remain preserved, as observed in less severe forms of the condition. Additionally, ictal fear and anger, often observed in MTLE, may utilize the same neural circuits as non-pathological emotional processing, suggesting a profound connection between the disorder and fundamental aspects of human emotion recognition (Reynders et al., 2005; Bartolomeil et al., 2002).

Considering both drug-sensitivity and side of the epileptic focus, we can identify 6 distinct groups lying along a spectrum. At one end there are the left mMTLE patients, whose performance statistically align with HC, followed by the left rMTLE, the right mMTLE, and the right rMTLE, all of whom perform statistically worse than HC; at the other end, the bilateral cases, who exhibit the worse performance. This distribution of groups reflects findings from Meletti et al. (2009), which shows that patients with a right focus generally have poorer outcomes than those with a left focus, and bilateral cases fare the worst. Interestingly, mMTLE patients with a left focus perform similarly to HC, but those with a right focus show deteriorated performance comparable to left drug-resistant patients. This pattern suggests that the right lateralization of MTLE has a greater impact on FER than seizure control. Overall, these observations indicate that MTLE patients without these two risk factors (right focus and drug-resistance) tend to maintain better FER performances.

### 4.2 Correlations with clinical variables

In addition to our primary objectives, we investigated the relationships between various clinical variables and performance on FER within our patient cohort. We assessed several parameters, including age at epilepsy onset, disease duration, number of ASMs, seizure frequency, history of FSs and MRI abnormalities, with a particular interest in MTS.

We observed a positive association between age at epilepsy onset and performance on EFT, indicating that an earlier onset of epilepsy was associated with lower scores on EFT. This aligns with other studies showing impaired FER in patients with early onset of epilepsy (Monti and Meletti, 2015; Bonora et al., 2011; McClelland et al., 2006; Meletti et al., 2014). For instance, Bonora et al. reported that patients with severe FER deficits had an earlier onset of epilepsy, whereas those with a later onset exhibited milder impairments (Bonora et al., 2011). Moreover, McClelland et al., studying a small cohort of patients with MTS, observed impairments in recognizing fearful expressions in patients with early onset, but not in those with late onset (McClelland et al., 2006). However, these findings are not consistently replicated across studies, likely due to the heterogeneity of patient cohorts or because many studies involve patients with relatively late onset of epilepsy (Bora and Meletti, 2016). Despite our cohort’s average age at onset being approximately 30 for mMTLE patients and 20 for rMTLE patients, a substantial proportion of them (46%) experienced epilepsy onset at or before 20 years of age, with 14% before 10 years and 10% before 5 years. It is possible that the substantial number of early-onset patients in our study supports this correlation.

An early onset of epilepsy may compromise social cognitive functions, particularly emotion recognition, by disrupting the maturation of circuits in fronto-temporo-limbic areas through epileptic discharges occurring during a critical developmental period which extends from childhood to adolescence. Indeed, previous studies have shown that the development of FER capabilities undergoes significant changes from early childhood to adulthood (Durand et al., 2007; Wade et al., 2006), even identifying two main stages, an initial one characterized by a uniform improvement across all emotions from ages 5 to 12, followed by a subsequent period in adolescence where the abilities continue to refine (Rodger et al., 2015).

The same multivariate analysis, repeated individually for the right and left foci, revealed that an early onset of the disease is correlated with poorer performances only in patients with a right focus. This finding is consistent with other data in the literature (Hlobil et al., 2008; Sedda et al., 2013) suggesting that there is a critical period during which the neural circuits responsible for emotion recognition mature, and that these circuits are predominantly represented in the right hemisphere, particularly in the mesial temporal lobe. An early onset of epilepsy in the right hemisphere could affect this maturation, impairing the ability to recognize emotions, irrespective of other subsequent factors such as pharmacological load, duration of the disease and seizure control.

The correlation between FSs and compromised FER is also consistent with findings in existing literature (Meletti et al., 2003; Cantalupo et al., 2013). For instance, Cantalupo et al. (2013) found that children with MTLE and a history of FSs exhibited decreased scores in FER compared to a control group. Our results confirm that FSs during childhood may impair the neural network responsible for processing basic emotions expressed through facial expressions.

Once again, conducting the same analysis separately for subjects with right and left foci, it emerged that a history of FSs is correlated with poorer FER performances in patients with a left but not in patients with right focus, the latter obtaining low scores regardless of the presence (39.77 ± 6.01) or absence (42.60 ± 8.66) of FSs. This might suggest that epileptogenic processes themselves in right MTLE could alter the maturation of FER circuits independently from FSs. Conversely, patients with a left focus might retain greater integrity of the right hemisphere, which is more important for the ability to recognize emotions, and are more affected by early generalized events such as FSs. It is indeed interesting to note how patients with a left focus and no FSs have relatively high average total scores (46.64 ± 4.50).

Finally, we found no correlation between poorer FER performance and the presence of MRI lesions compared to cryptogenic forms of MTLE. Even when selectively considering the presence of MTS, no statistically significant differences emerged in the total scores of the EFT between patients with and without MTS. However, conducting the same analysis separately for right and left foci, we found that MTS is associated with poorer performance in emotion recognition in patients with a left focus. It should also be noted that right patients displayed low scores regardless of the presence (41.85 ± 7.30) or absence (41.72 ± 9.02) of MTS, while left patients scored significantly higher in the absence of MTS (47.20 ± 4.55) compared to those with MTS (39.42 ± 5.08). This result aligns with other evidence in the literature (Hennion et al., 2015), and it is reasonable to consider that the otherwise preserved right hemisphere in left-sided epilepsy may be partially compromised by unilateral MTS, which could alter neuronal connections bilaterally (Lee et al., 2005; Camacho and Castillo, 2007; Pizzanelli et al., 2022). We hypothesize that MTS might involve processes of epileptogenesis occurring in early-life compared to other types of lesions, such as tumors or encephalitis, which appear later in life and therefore could be less capable of impacting the maturation of FER circuits (Meletti et al., 2003).

### 4.3 Strength and limitations of the study

To our knowledge this is the first study on FER in mild MTLE patients. Additionally, we have outlined a spectrum of MTLE that combines both the severity and lateralization of the epileptogenic focus. Despite the valuable insights provided by our study, several limitations should be acknowledged. First, the relatively small sample size, especially when subdivided by lateralization of the epileptic focus and disease severity, may limit the generalizability of our findings. Further studies with larger sample sizes are needed to confirm and expand upon our findings. Second, our study focused exclusively on patients with MTLE and did not include comparisons with other types of epilepsy. Future research should explore whether similar FER impairments are present in other epilepsy syndromes to determine the specificity of our findings to MTLE. Third, although we administered general cognitive assessments, which was normal for all patients, we did not include detailed neuropsychological tests of specific cognitive domains such as visuospatial abilities, attention, or memory. In this regard, an important consideration is whether the observed FER deficits are solely due to emotional processing impairments or are also influenced by deficits in visuospatial processing. Studies using the Rey-Osterrieth Complex Figure Test (ROCFT) or the Brief Visuospatial Memory Test—Revised (BVMT-R) have demonstrated changes in visual information processing in patients with TLE (McConley et al., 2006; Barr et al., 2004). This suggests that visuospatial deficits may contribute to the emotion recognition difficulties observed in our patients. Including comprehensive neuropsychological assessments in future studies would help disentangle the contributions of emotional and visuospatial deficits to FER impairments. Lastly, our cross-sectional design does not allow for causal inferences. Longitudinal studies could provide insights into how FER deficits evolve over time and in relation to disease progression or therapeutic interventions.

## 5 Conclusion

Mild MTLE exhibits compromised FER, albeit to a lesser extent than drug-resistant MTLE. Both right-side focus and drug resistance are risk factors for impairment in FER abilities in MTLE patients. Early onset of epilepsy in those with right focus could disrupt FER circuits, likely by interfering with a critical window for their maturation. In contrast, patients with left focus appear less vulnerable to damage directly caused by epilepsy. However, they tend to reach similar levels of impairment as right-focused individuals when other early-life factors, such as FSs or MTS, intervene.

Moreover, our findings suggest that the lateralization of the epileptic focus has a greater impact on FER impairment than seizure control. This is evident in patients with milder forms of MTLE, who exhibit FER deficits similar to their refractory counterparts when the focus is right, yet show performances comparable to healthy controls when the focus is left.

## Data Availability

The raw data supporting the conclusions of this article will be made available by the authors, without undue reservation.

## Supplementary material

SUPPLEMENTARY FIGURE S1 Boxplot of the comparison between the six groups in the EFT total score. EFT, Ekman Faces Test; HC, Healthy Controls; mMLTE, mild Mesial Temporal Lobe Epilepsy; rMTLE, resistant Mesial Temporal Lobe Epilepsy; **p* ≤ 0.05; ***p* ≤ 0.001.

## References

[ref1] Adolphs R. (2001). The neurobiology of social cognition. Curr. Opin. Neurobiol. 11, 231–239. doi: 10.1016/S0959-4388(00)00202-6, PMID: 11301245

[ref2] Barr W. Morrison C. Zaroff C. Devinsky O. (2004). Use of the brief visuospatial memory test-revised (BVMT-R) in neuropsychological evaluation of epilepsy surgery candidates. Epilepsy Behav. 5, 175–179. doi: 10.1016/j.yebeh.2003.12.010, PMID: 15123018

[ref3] Bartolomeil F. Guye M. Wendling F. Gavaret M. Regis J. Chauvel P. (2002). Fear, anger and compulsive behavior during seizure: involvement of large scale fronto-temporal neural networks. Epileptic Disord. 4, 235–241. doi: 10.1684/j.1950-6945.2002.tb00500.x, PMID: 12600809

[ref4] Beghi E. Giussani G. Nichols E. Abd-Allah F. Abdela J. Abdelalim A.. (2019). Global, regional, and national burden of epilepsy, 1990-2016: a systematic analysis for the global burden of disease study 2016. Lancet Neurol. 18, 357–375. doi: 10.1016/S1474-4422(18)30454-X, PMID: 30773428 PMC6416168

[ref5] Benuzzi F. Meletti S. Zamboni G. Calandra-Buonaura G. Serafini M. Lui F.. (2004). Impaired fear processing in right mesial temporal sclerosis: a fMRI study. Brain Res. Bull. 63, 269–281. doi: 10.1016/j.brainresbull.2004.03.005, PMID: 15196652

[ref6] Bonora A. Benuzzi F. Monti G. Mirandola L. Pugnaghi M. Nichelli P.. (2011). Recognition of emotions from faces and voices in medial temporal lobe epilepsy. Epilepsy Behav. 20, 648–654. doi: 10.1016/j.yebeh.2011.01.027, PMID: 21459049

[ref7] Bora E. Meletti S. (2016). Social cognition in temporal lobe epilepsy: a systematic review and meta-analysis. Epilepsy Behav. 60, 50–57. doi: 10.1016/j.yebeh.2016.04.024, PMID: 27179192

[ref8] Bressi C. Taylor G. Parker J. Bressi S. Brambilla V. Aguglia E.. (1996). Cross validation of the factor structure of the 20-item Toronto alexithymia scale: an Italian multicenter study. J. Psychosom. Res. 41, 551–559. doi: 10.1016/S0022-3999(96)00228-0, PMID: 9032718

[ref9] Broicher S. D. Kuchukhidze G. Grunwald T. Kramer G. Kurthen M. Jokeit H. (2012). “Tell me how do I feel” – emotion recognition and theory of mind in symptomatic mesial temporal lobe epilepsy. Neuropsychologia 50, 118–128. doi: 10.1016/j.neuropsychologia.2011.11.005, PMID: 22108441

[ref10] Camacho D. L. Castillo M. (2007). MR imaging of temporal lobe epilepsy. Semin. Ultrasound CT MR 28, 424–436. doi: 10.1053/j.sult.2007.09.005, PMID: 18074999

[ref11] Cantalupo G. Meletti S. Miduri A. Mazzotta S. Rios-Pohl L. Benuzzi F.. (2013). Facial emotion recognition in childhood: the effects of febrile seizures in the developing brain. Epilepsy Behav. 29, 211–216. doi: 10.1016/j.yebeh.2013.07.007, PMID: 23994831

[ref12] Costantini M. Musso M. Viterbori P. Bonci F. Del Mastro L. Garrone O.. (1999). Detecting psychological distress in cancer patients: validity of the Italian version of the hospital anxiety and depression scale. Support Care Cancer 7, 121–127. doi: 10.1007/s005200050241, PMID: 10335929

[ref13] DeKosky S. T. Heilman K. M. Bowers D. Valenstein E. (1980). Recognition and discrimination of emotional faces and pictures. Brain Lang. 9, 206–214. doi: 10.1016/0093-934X(80)90141-8, PMID: 7363065

[ref14] Durand K. Gallay M. Seigneuric A. Robichon F. Baudouin J. Y. (2007). The development of facial emotion recognition: the role of configural information. J. Exp. Child Psychol. 97, 14–27. doi: 10.1016/j.jecp.2006.12.001, PMID: 17291524

[ref15] Edwards M. Stewart E. Palermo R. Lah S. (2017). Facial emotion perception in patients with epilepsy: a systematic review with meta-analysis. Neurosci. Biobehav. Rev. 83, 212–225. doi: 10.1016/j.neubiorev.2017.10.013, PMID: 29045812

[ref16] Fisher R. S. Acevedo C. Arzimanoglou A. Bogacz A. Cross J. H. Elger C. E.. (2014). ILAE official report: a practical clinical definition of epilepsy. Epilepsia 55, 475–482. doi: 10.1111/epi.12550, PMID: 24730690

[ref17] Fisher R. S. Cross J. H. French J. A. Higurashi N. Hirsch E. Jansen F. E.. (2017). Operational classification of seizure types by the international league against epilepsy: position paper of the ILAE Commission for Classification and Terminology. Epilepsia 58, 522–530. doi: 10.1111/epi.13670, PMID: 28276060

[ref18] Gainotti G. (1983). Emotions and hemispheric lateralization. Review of the literature. Encéphale 9, 345–364, PMID: 6368198

[ref19] Golouboff N. Fiori N. Delalande O. Fohlen M. Dellatolas G. Jambaque I. (2008). Impaired facial expression recognition in children with temporal lobe epilepsy: impact of early seizure onset on fear recognition. Neuropsychologia 46, 1415–1428. doi: 10.1016/j.neuropsychologia.2007.12.019, PMID: 18249422

[ref20] Hennion S. Szurhaj W. Duhamel A. Lopes R. Tyvaert L. Derambure P.. (2015). Characterization and prediction of the recognition of emotional faces and emotional bursts in temporal lobe epilepsy. J. Clin. Exp. Neuropsychol. 37, 931–945. doi: 10.1080/13803395.2015.1068280, PMID: 26332173

[ref21] Hlobil U. Rathore C. Alexander A. Sarma S. Radhakrishnan K. (2008). Impaired facial emotion recognition in patients with mesial temporal lobe epilepsy associated with hippocampal sclerosis (MTLE-HS): side and age at onset matters. Epilepsy Res. 80, 150–157. doi: 10.1016/j.eplepsyres.2008.03.018, PMID: 18468867

[ref22] Jasionis A. Puteikis K. Mameniskiene R. (2021). The impact of social cognition on the real-life of people with epilepsy. Brain Sci. 11:887. doi: 10.3390/brainsci11070877, PMID: 34209039 PMC8301878

[ref23] Juncai S. Jing Z. Shi R. (2017). Differentiating recognition for anger and fear facial expressions via inhibition of return. J. Psychol. Cogn. 2:02. doi: 10.35841/psychology-cognition.2.1.10-16

[ref24] Kobiella A. Grossmann T. Reid V. Striano T. (2008). The discrimination of angry and fearful facial expressions in 7-month-old infants: an event-related potential study. Cognit. Emot. 22, 134–146. doi: 10.1080/02699930701394256

[ref25] Kuchukhidze G. Unterberger I. Schmid E. Zamarian L. Siedentopf C. M. Koppelstaetter F.. (2021). Emotional recognition in patients with mesial temporal epilepsy associated with enlarged amygdala. Front. Neurol. 12:803787. doi: 10.3389/fneur.2021.803787, PMID: 35126298 PMC8815259

[ref26] Kwan P. Arzimanoglou A. Berg A. T. Brodie M. J. Allen Hauser W. Mathern G.. (2010). Definition of drug resistant epilepsy: consensus proposal by the ad hoc task force of the ILAE commission on therapeutic strategies. Epilepsia 51, 1069–1077. doi: 10.1111/j.1528-1167.2009.02397.x, PMID: 19889013

[ref27] Labate A. Gambardella A. Andermann E. Aguglia U. Cendes F. Berkovic S. F.. (2011). Benign mesial temporal lobe epilepsy. Nat. Rev. Neurol. 7, 237–240. doi: 10.1038/nrneurol.2010.212, PMID: 21263461

[ref28] Lee S. K. Kim D. W. Kim K. K. Chung C. K. Song I. C. Chang K. H. (2005). Effect of seizure on hippocampus in mesial temporal lobe epilepsy and neocortical epilepsy: an MRS study. Neuroradiology 47, 916–923. doi: 10.1007/s00234-005-1447-8, PMID: 16158277

[ref29] Marco-Garcia S. Ferrer-Quintero M. Usall J. Ochoa S. Del Cacho N. Huerta-Ramos E. (2019). Facial emotion recognition in neurological disorders: a narrative review. Rev. Neurol. 69, 207–219. doi: 10.33588/rn.6905.2019047, PMID: 31364150

[ref30] McClelland S. 3rd Garcia R. E. Peraza D. M. Shih T. T. Hirsch L. J. Hirsch J.. (2006). Facial emotion recognition after curative nondominant temporal lobectomy in patients with mesial temporal sclerosis. Epilepsia 47, 1337–1342. doi: 10.1111/j.1528-1167.2006.00557.x, PMID: 16922878

[ref31] McConley R. Martin R. Banos J. Blanton P. Faught E. (2006). Global/local scoring modifications for the Rey-Osterrieth complex figure: relation to unilateral temporal lobe epilepsy patients. J. Int. Neuropsychol. Soc. 12, 383–390. doi: 10.1017/S1355617706060413, PMID: 16903130

[ref32] Meletti S. Benuzzi F. Cantalupo G. Rubboli G. Tassinari C. A. Nichelli P. (2009). Facial emotion recognition impairment in chronic temporal lobe epilepsy. Epilepsia 50, 1547–1559. doi: 10.1111/j.1528-1167.2008.01978.x, PMID: 19175397

[ref33] Meletti S. Benuzzi F. Rubboli G. Cantalupo G. Stanzani Maserati M. Nichelli P.. (2003). Impaired facial emotion recognition in early-onset right mesial temporal lobe epilepsy. Neurology 60, 426–431. doi: 10.1212/WNL.60.3.426, PMID: 12578923

[ref34] Meletti S. Picardi A. De Risi M. Monti G. Esposito V. Grammaldo L. G.. (2014). The affective value of faces in patients achieving long-term seizure freedom after temporal lobectomy. Epilepsy Behav. 36, 97–101. doi: 10.1016/j.yebeh.2014.05.002, PMID: 24892756

[ref35] Monti G. Meletti S. (2015). Emotion recognition in temporal lobe epilepsy: a systematic review. Neurosci. Biobehav. Rev. 55, 280–293. doi: 10.1016/j.neubiorev.2015.05.009, PMID: 25999121

[ref36] Natale M. Gur R. E. Gur R. C. (1983). Hemispheric asymmetries in processing emotional expressions. Neuropsychologia 21, 555–565. doi: 10.1016/0028-3932(83)90011-8, PMID: 6646407

[ref37] Pizzanelli C. Pesaresi I. Milano C. Cecchi P. Fontanelli L. Giannoni S.. (2022). Distinct limbic connectivity in left and right benign mesial temporal lobe epilepsy: evidence from a resting state functional MRI study. Front. Neurol. 13:943660. doi: 10.3389/fneur.2022.943660, PMID: 36247782 PMC9558280

[ref38] Qi L. Zhao J. Zhao P. Zhang H. Zhong J. Pan P.. (2022). Theory of mind and facial emotion recognition in adults with temporal lobe epilepsy: a meta-analysis. Front. Psych. 13:976439. doi: 10.3389/fpsyt.2022.976439, PMID: 36276336 PMC9582667

[ref39] Reynders H. J. Broks P. Dickson J. M. Lee C. E. Turpin G. (2005). Investigation of social and emotion information processing in temporal lobe epilepsy with ictal fear. Epilepsy Behav. 7, 419–429. doi: 10.1016/j.yebeh.2005.07.013, PMID: 16176889

[ref40] Rodger H. Vizioli L. Ouyang X. Caldara R. (2015). Mapping the development of facial expression recognition. Dev. Sci. 18, 926–939. doi: 10.1111/desc.12281 25704672

[ref41] Santangelo G. Siciliano M. Pedone R. Vitale C. Falco F. Bisogno R.. (2015). Normative data for the Montreal cognitive assessment in an Italian population sample. Neurol. Sci. 36, 585–591. doi: 10.1007/s10072-014-1995-y, PMID: 25380622

[ref42] Sartori G. Colombo L. Vallar G. Rusconi M. L. Pinarello A. (1995). TIB: Test di Intelligenza Breve per la valutazione del quoziente intellettivo attuale e pre-morboso. Giornale dell'Ordine degli Psicologi. 4, 1–24.

[ref43] Scheffer I. E. Berkovic S. Capovilla G. Connolly M. B. French J. Guilhoto L.. (2017). ILAE classification of the epilepsies: position paper of the ILAE Commission for Classification and Terminology. Epilepsia 58, 512–521. doi: 10.1111/epi.13709, PMID: 28276062 PMC5386840

[ref44] Sedda A. Rivolta D. Scarpa P. Burt M. Frigerio E. Zanardi G.. (2013). Ambiguous emotion recognition in temporal lobe epilepsy: the role of expression intensity. Cogn. Affect. Behav. Neurosci. 13, 452–463. doi: 10.3758/s13415-013-0153-y, PMID: 23430725

[ref45] Steiger B. K. Jokeit H. (2017). Why epilepsy challenges social life. Seizure 44, 194–198. doi: 10.1016/j.seizure.2016.09.008, PMID: 27756511

[ref46] Tanaka A. Akamatsu N. Yamano M. Nakagawa M. Kawamura M. Tsuji S. (2013). A more realistic approach, using dynamic stimuli, to test facial emotion recognition impairment in temporal lobe epilepsy. Epilepsy Behav. 28, 12–16. doi: 10.1016/j.yebeh.2013.03.022, PMID: 23648274

[ref47] Wade A. M. Lawrence K. Mandy W. Skuse D. (2006). Charting the development of emotion recognition from 6 years of age. J. Appl. Stat. 33, 297–315. doi: 10.1080/02664760500445756

[ref48] Walpole P. Isaac C. L. Reynders H. J. (2008). A comparison of emotional and cognitive intelligence in people with and without temporal lobe epilepsy. Epilepsia 49, 1470–1474. doi: 10.1111/j.1528-1167.2008.01655.x, PMID: 18479383

