# Supplementary figures and images for: Exploring the role of epileptic focus lateralization on facial emotion recognition in the spectrum of mesial temporal lobe epilepsy
Source: Front Syst Neurosci. 2025 Jan 6;18:1491791. doi: 10.3389/fnsys.2024.1491791 (PMC11743968; doi:10.3389/fnsys.2024.1491791)

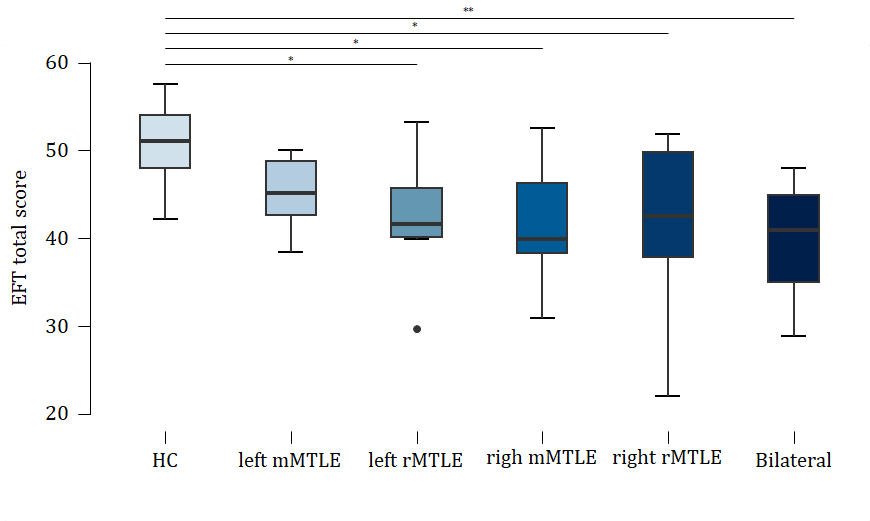

Supplement: SUPPLEMENTARY FIGURE S1 — Boxplot of the comparison between the six groups in the EFT total score. EFT, Ekman Faces Test; HC, Healthy Controls; mMLTE, mild Mesial Temporal Lobe Epilepsy; rMTLE, resistant Mesial Temporal Lobe Epilepsy; *p ≤ 0.05; **p ≤ 0.001. [file Image_1.JPEG]
